# Seroprevalence of SARS-CoV-2 antibodies in healthcare workers at a London NHS Trust

**DOI:** 10.1017/ice.2020.402

**Published:** 2020-08-04

**Authors:** Joseph J. Grant, Stephanie M.S. Wilmore, Naina S. McCann, Owain Donnelly, Rebecca W.L. Lai, Matthew J. Kinsella, Helena L. Rochford, Trupti Patel, Michael C. Kelsey, Julie A. Andrews

**Affiliations:** Department of Microbiology, Whittington Health NHS Trust, London, United Kingdom

## Abstract

Healthcare workers (HCWs) have a theoretically increased risk of contracting severe acute respiratory coronavirus virus 2 (SARS-CoV-2) given their occupational exposure. We tested 2,167 HCWs in a London Acute Integrated Care Organisation for antibodies to SARS-CoV-2 in May and June 2020 to evaluate seroprevalence. We found a seropositivity rate of 31.6% among HCWs.

The United Kingdom has experienced a large outbreak of coronavirus disease 2019 (COVID-19) since the first cases were diagnosed in February 2020. Mounting evidence from international sources suggests that healthcare workers (HCWs) have been disproportionately affected despite the use of personal protective equipment (PPE).^1^ Reverse transcription polymerase chain reaction (RT-PCR) screening of symptomatic HCWs has provided an estimate of infection rates but the low sensitivity of the test and logistical limitations of screening individuals in self-isolation has hindered our understanding.^2^ The picture is clouded further by our limited knowledge of asymptomatic infections in this population.^3^

Serological testing provides an opportunity to further our understanding. Commercially available serological tests have demonstrated significantly increased sensitivity relative to RT-PCR and are able to provide retrospective evidence of infection.^2,4^

Whittington Health NHS Trust is an Acute Integrated Care Organisation in London, UK. It employs ~4,000 people spread across a 360-bed acute-care site, emergency department (ED), and 30 community sites. Herein, we present data from 2004 HCWs who were tested for SARS-CoV-2 antibodies at the Trust from May 15 to June 5, 2020.

## Methods

All Whittington Health employees were invited to self-refer for a test via departmental managers and Trust-wide communication emails. All were eligible regardless of job or whether they had experienced a COVID-19–like illness.

Data were collected on age, ethnicity, history, and date of COVID-19–like illness, if any, and whether the HCW had previously had an RT-PCR test. Each HCW categorized themselves by their workplace and extent of patient contact during the pandemic peak (March–May 2020).

Samples were tested using the Elecsys Anti–SARS-CoV-2 assay (Roche Diagnostics, Basel, Switzerland). This electro-chemiluminescence immunoassay is a combined assay for IgG and IgM. A qualitative result of “SARS-CoV-2 antibody detected” or “SARS-CoV-2 antibody not-detected” was produced. Validation by Public Health England (PHE) showed a sensitivity of 84%–100% and a specificity of 99.8%.^4^

### Data analysis

Data were entered into the laboratory information management system WinPath. A Microsoft Excel spreadsheet was then populated from this and were analyzed using Stata version 12.1 software (StataCorp, College Station, TX). We evaluated differences in proportions with χ^2^ tests with continuity correction (significance threshold, *P* < .05).

### Ethics

This report describes and analyses the care provided to Whittington Health staff members. Following discussion with the Trust’s research leader and application of the NHS Health Research Authority algorithm, it was decided that no ethical approval was required for this evaluation.

## Results

Between May 15 and June 5, 2020, we tested 2,167 HCWs; we excluded 163 due to incomplete data and therefore included 2004 HCWs in the final analysis. Antibodies were detected in 634 HCWs (31.64%). The ages of those tested ranged from 18 to 73 years; the mean age was 40.21 (95% CI, 39.7–40.7).

### Degree of clinical contact and working environment

HCWs were asked to identify which working environment and ward type best matched their degree of patient contact during the pandemic (Tables 1 and 2).

**Table 1.** Seropositivity to SARS-CoV-2 in HCWs at Whittington Health NHS Trust Grouped by Working Environment and Degree of Patient Contact During the Peak of the Pandemic (March–May 2020)^a^

Exposure Type	Example Job Role	Ab Detected Within the Given Exposure Type, n/N (%)	Ab Detected in All Other Exposure Types Combined, n/N (%)	*P* Value^b^
I work in a clinical environment and have prolonged direct contact with patients	Doctor, nurse, physiotherapist, porter	467/1,345 (34.7)	167/659 (25.3)	**<.005**
I work in a clinical environment and have less/no patient contact	Pharmacist, domestic staff	59/197 (30.0)	575/1,807 (31.8)	.592
I work in a nonclinical environment and have prolonged direct contact with patients	District nurses and rapid response teams	27/108 (25.0)	607/1,896 (32.0)	.127
I work in a nonclinical environment and have minimal to no patient contact	Office or laboratory based staff	77/341 (22.6)	557/1,663 (33.5)	**<.005**
I have been working from home/shielding	Anyone working from home	4/13 (30.8)	630/1,991 (31.6)	.946

Note. Ab, anti–SARS-CoV-2 antibody.

a Seropositivity in each exposure type is compared to the seropositivity in the population of all the other exposure types combined.

b χ^2^ (1) each exposure type compared to all other types. Bold *P* value indicates significance.

**Table 2.** Seropositivity to SARS-CoV-2 in HCWs at Whittington Health NHS Trust Grouped by Ward Type During the Peak of the Pandemic (March–May 2020)^a^

Ward Type	Ab Positive Within the Given Ward Type, n/N (%)	Ab Positive in All Other Ward Types Combined, n/N (%)	*P* Value^b^
COVID-19 ward with CPAP	84/200 (42.0)	550/1,804 (30.5)	**.001**
COVID-19 ward without CPAP(including ED)	175/424 (41.3)	459/1,580 (29.1)	**<.005**
Operating Theatres	43/128 (33.6)	591/1,876 (31.5)	.623
Non-COVID-19 ward (including maternity and neonatal unit)	82/284 (28.9)	552/1,720 (32.1)	.280
Non-ward environment (any environment not listed)	206/792 (26.0)	428/1,212 (35.3)	**<.005**
ITU	44/176 (25.0)	590/1,828 (32.3)	**.047**

Note. Ab, anti–SARS-CoV-2 antibody; CPAP, continuous positive airway pressure; ED, emergency department; ITU, intensive treatment unit.

a Seropositivity in each ward type is compared to the seropositivity in the population of all the other ward types combined.

b χ^2^ (1) each exposure type compared to all other types. Bold *P* value indicates significance.

Seropositivity was highest among staff working in a clinical environment with direct patient contact (34.7%). This rate was significantly higher than that of all other exposure types combined (*P* < .005). Seropositivity was lowest among those working in nonclinical environments without patient contact (22.6%), this rate was significantly lower than that of all other exposure types combined (*P* < .005).

When analysed by ward type, seroprevalence was significantly higher on COVID-19 wards (COVID-19 ward with continuous positive airway pressure (CPAP) = 42.0%, (p = .001), COVID19 ward without CPAP = 41.3%, (p < .005)) compared to all other ward types combined. Seroprevalence was lowest in the intensive treatment unit (ITU; 25.0%). This rate was significantly lower than that of all other ward types combined (*P* = .047).

In a subgroup analysis of seropositivity rates of HCWs working on COVID-19 wards, no statistically significant difference in seroprevalence was found between the wards where CPAP (n = 624) was used and those where it was not (*P* = .864).

### History of illness

Of those tested, 1,031 of 2,004 (51.5%) self-described a COVID-19–like illness, with typical symptoms listed on the request form. In the seropositive group, 490 of 634 (77.3%) reported a COVID-19–like illness, 139 of 634 (21.9%) did not report COVID-19 symptoms, and 5 of 634 (0.8%) did not state their status.

### RT-PCR positivity

A small subset of those tested (285 of 2004, 14.2%) were tested by RT-PCR for SARS-CoV-2 at the time of their reported illness. Of those who tested positive by RT-PCR (71 of 285, 24.9%), most (68 of 71, 95.8%) had detectable antibodies. Of those who tested negative by RT-PCR, 57 of 214 (26.6%) had detectable antibodies.

## Discussion

The seropositivity of HCWs at Whittington Health NHS Trust is nearly double that of the PHE estimate for London’s general population (31.0% vs 17.5%).^5^ Contributing factors to increased HCW exposure may include patient-to-HCW transmission, HCW-to-HCW transmission, or increased contact exposure during travel to work. Concerns over HCW-to-HCW transmission have been raised because space constraints in many NHS Trusts prevent compliance with social distancing guidelines. This factor, in addition to evidence of nosocomial transmission, have prompted recent changes to the UK government’s policy on the use of masks in healthcare settings.^6^

Few studies to date have assessed HCW seroprevalence of SARS-CoV-2 antibodies. International reports show that seroprevalence varies widely, with rates as high as 33% in New York and as low as 1.6% in Essen, Germany.^7,8^ This range may reflect the difference in disease burden in these cities.

In our Trust, working on a COVID-19 ward was associated with a higher rate of seropositivity. However, HCWs on COVID-19 wards where aerosol-generating procedures (AGPs) were performed did not have significantly higher rates of seropositivity compared to other COVID-19 wards. The reasons for this are likely multifactorial, but contributing factors could include the use of enhanced PPE for AGPs or a greater awareness of the need for infection control in these areas. We included the ED as a “COVID-19 ward without CPAP” because only a small proportion of patients there underwent AGPs (intubation and CPAP).

Surprisingly, ITU HCWs had the lowest rates of seropositivity. This finding may relate to the fact that once intubated, the patients are ventilated on a closed circuit. The natural history of COVID-19 may also contribute; those who required ITU admission were often admitted on or around day 10^9^ of their illness, by which point they were less likely to be infectious.^10^ Differences in PPE use may also be relevant. Enhanced PPE for AGPs was used at all times within the ITU. PPE used in the Trust is outlined in the Supplementary Material (online).

Among our seropositive staff, 21.9% reported no prior COVID-19–like illness. This finding suggests that a significant proportion of COVID-19 infections in our cohort were pauci-symptomatic or asymptomatic. These individuals would not have been self-isolating when infected and could have acted as vectors for nosocomial transmission. The PHE policy has since changed and face masks are now worn in any area where social distancing is not possible, helping to mitigate this risk.

The limitations of this report include a potential selection bias because HCWs were invited to self-refer for testing. No data on sex of HCWs were collected, and ethnicity data were collected separately, preventing comparative analyses.

Despite being a single-center evaluation, our results suggest that HCW seroprevalence may be higher than expected. Further studies are needed to assess whether these results are representative of other UK hospitals. Should this be the case, a re-evaluation of infection control and social distancing measures currently in place within NHS hospitals may be urgently required.

## Supplementary material

For supplementary material accompanying this paper visit http://dx.doi.org/10.1017/ice.2020.402. click here to view supplementary material
