# Supplementary material for: Seroprevalence of SARS-CoV-2 antibodies in healthcare workers at a London NHS Trust
Source: Infect Control Hosp Epidemiol. 2020 Aug 4:1–3. doi: 10.1017/ice.2020.402 (PMC7438618; doi:10.1017/ice.2020.402)
Supplement: Supplementary file 1 [file S0899823X2000402Xsup001.docx]

| **PPE Used** | **Scenario used** |
| --- | --- |
| - Fluid resistant surgical mask - Eye protection if at risk of splashing into eyes - Gloves - Plastic apron | - Before 1^st^ April – When within 2m of a patient with a Positive COVID-19 PCR test or significant clinical suspicion of COVID-19 - After 1^st^ April – When caring for any patient in any clinical environment |
| - FFP3 respirator mask - Eye protection - Gloves - Long sleeve disposable gown | - Any aerosol generating procedure in a confirmed or suspected COVID-19 patient   Or   - At any time when in ITU |
| - Fluid resistant surgical mask | - After 15^th^ June – at all times when unable to maintain a 2m distance from other people. |

**Supplementary Material**

**Supplementary Table 1** – Components of, and scenarios in which PPE were used at Whittington Health NHS Trust between March and June 2020. These are in line with Public Health England guidance. No PPE shortages were experienced at the trust.
